# Supplementary material for: Chinese herbal medicine for post-viral fatigue: A systematic review of randomized controlled trials
Source: PLoS One. 2024 Mar 21;19(3):e0300896. doi: 10.1371/journal.pone.0300896 (PMC10956782; doi:10.1371/journal.pone.0300896)
Supplement: S2 Appendix — (DOCX) [file pone.0300896.s002.docx]

1、CNKI

TKA=('病毒'+'病毒感染'+'乙型肝炎'+'hepatitis b'+'乙肝'+'乙型病毒肝炎'+'慢性乙型肝炎'+'慢乙型肝炎'+'病毒性乙型肝炎'+'乙型病毒性肝炎'+'病毒性乙肝'+'病毒性肝炎乙型'+'慢性乙型肝炎患者'+'慢性病毒性乙肝'+'慢性乙型病毒性肝炎'+'慢乙肝'+'慢性乙肝肝炎'+'慢性乙肝'+'慢性乙型病毒肝炎'+'慢性病毒性乙型肝炎'+'慢性乙肝病毒性肝炎'+'甲型病毒性肝炎'+'hepatitis a'+'甲肝'+'甲型肝炎'+'丙型肝炎'+'hepatitis c'+'丙肝'+'丙型病毒性肝炎'+'慢性丙型肝炎'+'丙型病毒肝炎'+'慢性病性肝炎'+'丁型肝炎'+'hepatitis d'+'丁型病毒性肝炎'+'丁肝'+'戊型肝炎'+'hepatitis e'+'戊肝'+'戊型病毒性肝炎'+'流行性乙型脑炎'+'大脑炎'+'乙脑'+'乙型脑炎'+'手足口病'+'hand-foot-mouth disease'+'手足口症'+'手足口疾病'+'手足口综合征'+'流感'+'Flu'+'Influenza'+'流行性感冒'+'H1N1'+'禽流感'+'H7N9'+'HIV'+'hiv感染'+'hiv'+'艾滋'+'AIDS'+'带状疱疹'+'带状性疱疹'+'蜘蛛疮'+'蛇串疮'+'水痘'+'covid-19'+'病毒性肺炎'+'COVID-19'+'新冠肺炎'+'新型冠状病毒肺炎'+'传染性单核细胞增多症'+'infectious mononucleosis syndrome'+'腺热'+'非典型肺炎'+'atypical pneumonia'+'atypical pneumoniae'+'Sars'+'重症急性呼吸综合征'+'严重急性呼吸道综合症'+'急性呼吸综合征'+'传染性非典型肺炎'+'严重急性呼吸器官综合征'+'严重急性呼吸系统综合症'+'严重急性呼吸道症候群'+'非典型性肺炎'+'非典'+'严重急性呼吸综合征'+'严重急性呼吸综合症'+'严重急性呼吸道综合征'+'流行性腮腺炎'+'麻疹'+'风疹'+'巨细胞病毒感染'+'肠道病毒感染'+'脊髓灰质炎'+'柯萨奇病毒感染'+'病毒感染性腹泻'+'肾综合征出血热'+'流行性出血热'+'登革热'+'登革出血热') AND TKA=('慢性疲劳综合症'+'chronic fatigue syndrome'+'慢性疲劳综合征'+'乏力'+'疲劳'+'疲乏'+'劳累'+'缓解期'+'恢复期') AND TKA=('中医药'+'中药'+'中草药'+'中医'+'中成药'+'汤'+'丸'+'散'+'膏'+'丹') AND TKA='随机'

2、Pubmed:

#1： viral or viruses or serious acute respiratory syndrome or SARSCoV or covid-19 or H1N1 or Influenza or H7N9 or hepatitis a or hepatitis b or hepatitis c or hepatitis d or hepatitis e or HIV or AIDS or [Infectious Mononucleosis](https://www.ncbi.nlm.nih.gov/mesh/68007244) [Herpes Zoster](https://www.ncbi.nlm.nih.gov/mesh/68006562) or Hand Foot and Mouth Disease or [Encephalitis Japanese](https://www.ncbi.nlm.nih.gov/mesh/68004672) or Cytomegalovirus or Infectious Mononucleosis or Hemorrhagic Fever with Renal Syndrome or Enterovirus or Dengue

#2：post-viral fatigue or post viral fatigue or chronic fatigue or fatigue or chronic fatigue syndrome or convalescence

#3：traditional Chinese medicine or herbal medicine or Chinese patent medicine

#4："randomized controlled trial"[Publication Type] OR "controlled clinical trial"[Publication Type] OR "randomized"[Title/Abstract] OR "placebo"[Title/Abstract] OR "randomly"[Title/Abstract] OR "Trial"[Title/Abstract] OR "groups"[Title/Abstract]

#5：#1 or #2

#6：#5 and #3 and #4
